# Prevalence of sarcopenia among older adults in the Middle East countries: a meta-analysis

**DOI:** 10.3389/fpubh.2026.1968834

**Published:** 2026-10-23

**Authors:** Bader A. Alqahtani, Norah A. Alhwoaimel, Bashayer A. AlHarbi, Ahmad A. Alsayer, Aqeel M. Alenazi

**Affiliations:** 1 Department of Health and Rehabilitation Sciences, Prince Sattam Bin Abdulaziz University, Al-Kharj, Saudi Arabia; 2 Saudi Medical Appointments and Referrals Center, Ministry of Health, Riyadh, Saudi Arabia; 3 School of Education, Taibah University, Madinah, Saudi Arabia

**Keywords:** Middle East, muscle mass, older adults, sarcopenia, systematic review

## Abstract

This systematic review aims to determine the prevalence of sarcopenia among community-dwelling older adults in Middle Eastern countries.

**Methods:**

A meta-analysis was conducted. A computerized systematic literature search of PubMed, Web of Science, and SCOPUS from inception to September 2025 was performed to capture all possible relevant titles. Inclusion criteria included all primary studies conducted in Middle Eastern countries on Sarcopenia in community-dwelling older adults aged 60 years and older.

**Results:**

A total of 11 studies were included in the study. Random-effects meta-analysis of 11 studies indicated a pooled prevalence rate of sarcopenia among older adults in the Middle East was 35% (95% CI, 23 to 48, *I*^2^ = 99.3%), with a higher sarcopenia rate among women (28%) compared to men (26%). The Egger's regression test for detecting publication bias did not detect any evidence of publication bias in the sample of included studies.

**Conclusions:**

This meta-analysis shows that the overall prevalence of sarcopenia in older adults in the Middle East is relatively high (35%), with a higher sarcopenia rate among women (28%) compared to men (26%). These findings highlight that sarcopenia is a significant public health concern requiring a national public health strategy to prevent its progression and associated complications.

## 1 Introduction

Sarcopenia is a geriatric condition defined as a progressive skeletal muscle disorder characterized by a gradual loss of muscle mass, a decline in muscle strength, and a limitation in physical performance. (1). This condition leads to adverse outcomes such as an increased risk of falling, musculoskeletal disorders, physical disability, and mortality. It is officially recognized as a muscle disease by the International Classification of Diseases (ICD-10: M62). (2). Sarcopenia is prevalent among older populations, with rates ranging from 10% to 27% globally, depending on the definitions used, including European Working Group on Sarcopenia in Older People (EWGSOP2), Asian Working Group for Sarcopenia (AWGS), International Working Group on Sarcopenia (IWGS), and Foundation for the National Institutes of Health (FNIH) (3). Sarcopenia is probable when low muscle strength is detected, and as severe when the individual has decreased muscle strength, low muscle mass, and limited physical performance (4). Identification begins with the SARC-F questionnaire to screen at-risk individuals, followed by confirming the diagnosis through the evaluation of muscle quantity or quality using techniques such as dual-energy X-ray absorptiometry (DXA) or bioelectrical impedance analysis (BIA). Severity is determined by assessing physical performance through tests such as gait speed, Short Physical Performance Battery (SPPB), or Timed Up and Go (TUG) tests (5). The staging of sarcopenia includes possible sarcopenia (low muscle strength or physical performance), sarcopenia (low muscle mass plus low muscle strength or decreased performance), and severe sarcopenia (all three criteria).

Several risk factors and diseases are found to be associated with a high risk of sarcopenia, such as aging, malnutrition, sedentary behavior, obesity, diabetes mellitus, cardiovascular diseases, chronic kidney disease, and other chronic diseaases (6). Aging is considered the primary risk factor and a key factor used to diagnose sarcopenia. This age-related muscle deterioration is influenced by multiple biological changes, such as hormonal alterations (e.g., decreases in growth hormone and testosterone), chronic low-grade inflammation, neuromuscular junction degeneration, and mitochondrial dysfunction (7). An unbalanced diet with frequently consumed nutritionally inadequate meals—such as those high in carbohydrates but low in protein, fruits, and vegetables—had nearly double the odds of developing sarcopenia (8). Sedentary lifestyle behaviors have been increasingly recognized as key modifiable risk factors in the development and progression of sarcopenia among older adults (9). Obesity is also defined as a risk factor for sarcopenia, particularly in older adults, which is associated with a decrease in muscle mass and strength. This relationship is attributed to several factors, including chronic low-grade inflammation, insulin resistance, and hormonal changes, all of which can impair muscle function and contribute to the development of sarcopenia. Being chronically ill is linked to an increased risk of sarcopenia. For instance, diabetes is identified as a significant risk factor for sarcopenia through multiple interconnected mechanisms. Chronic hyperglycemia and insulin resistance, hallmark features of type 2 diabetes, contribute to accelerated muscle mass loss and functional decline. Participants with heart disease were also at an elevated risk of developing sarcopenia compared to those without heart disease. Chronic kidney disease (CKD) was associated with an increased risk of sarcopenia, consistent with existing literature that reports a global prevalence of sarcopenia in CKD patients ranging from 24.5% across all disease stages. Arthritis was also found to be significantly associated with sarcopenia risk, highlighting the impact of joint diseases on muscle health (10).

Significant regional disparities in the prevalence of sarcopenia have been reported, with individuals residing in rural areas generally at higher risk compared to their urban counterparts, partly due to environmental and socioeconomic differences (11). Rural populations often face economic challenges, including lower income levels and limited access to healthcare services, which can impede early diagnosis and management of sarcopenia. Inadequate nutrition, particularly insufficient protein intake, is more prevalent in rural areas. This nutritional deficiency contributes to muscle mass loss and functional decline. Differences in occupational and recreational physical activities between urban and rural settings influence muscle health. While rural residents may engage in more labor-intensive work, the lack of structured exercise programs and facilities can limit overall physical activity beneficial for muscle maintenance. In addition to the higher rates of chronic conditions such as cardiovascular diseases and diabetes in rural areas exacerbate the risk of sarcopenia, as these illnesses can accelerate muscle degradation (11). In the Middle East context, there are unique regional factors (12–14). Higher body mass index (BMI) was observed in individuals with Metabolic Syndrome (MetS), and when combined with pre-sarcopenia, it correlated with adverse cardiometabolic profiles (15). Higher rates of obesity, physical inactivity, and diabetes prevalence among Saudi Arabia, Iran, Iraq, Lebanon, Jordan, and UAE populations were highlighted (16–20). The growing population of older adults in the Middle East, is also a special consideration in the context. There is a significant increase in the aging population, and this shift is attributed to declining fertility rates and increased life expectancy across the region. This rapid demographic change poses potential challenges for social and economic structures, particularly concerning healthcare services, pension systems, and workforce dynamics, underscoring the importance of proactive policy planning to address the impending needs of an ageing population in the Middle East countries (21).

Several studies were conducted to determine the prevalence of sarcopenia among healthy older populations in Western and Eastern regions, but a few were conducted in the Middle East. In the Middle East context, several regional characteristics may increase the relevance of sarcopenia as a public health concern. High prevalence of obesity, physical inactivity, and diabetes, along with a rapidly aging population, may contribute to increased vulnerability to sarcopenia in this region. These contextual factors underscore the importance of establishing reliable prevalence estimates of sarcopenia among older adults in the Middle East. Therefore, this systematic review with meta-analysis aims to determine the prevalence of sarcopenia among community-dwelling older adults in Middle Eastern countries. In addition, this review provides a descriptive overview of factors reported in the literature that may be associated with sarcopenia in this region. However, these factors were not quantitatively synthesized due to variability and limited reporting across studies.

## 2 Methods

### 2.1 Protocol

The PROSPERO registration number for the systematic review protocol is CRD420250632088. The results of this systematic review and meta-analysis are presented in accordance with PRISMA criteria (21).

### 2.2 Eligibility criteria

Inclusion criteria were as follows: observational studies, including cross-sectional, cohort, and longitudinal studies, that assessed sarcopenia in older adults using established or clearly defined methods. Eligible studies included those applying consensus-based diagnostic criteria, such as the original European Working Group on Sarcopenia in Older People consensus (EWGSOP1) or its revised framework (EWGSOP2), Asian Working Group for Sarcopenia (AWGS), International Working Group on Sarcopenia (IWGS), or Foundation for the National Institutes of Health (FNIH), validated screening tools (e.g., SARC-F), or explicitly defined indices based on measures of muscle mass, muscle strength, and/or physical performance.

Studies conducted in the Middle East countries. Studies published in English-language only were included. We excluded any study that has examined Sarcopenia in older adults with a specific disease or condition. Studies focusing on older adults with specific diseases or clinical conditions were excluded to minimize potential confounding, as many chronic conditions are independently associated with sarcopenia and may inflate prevalence estimates. This restriction was applied to improve the validity and comparability of prevalence estimates in the general community-dwelling older adult population.

### 2.3 Search strategy

A computerized systematic literature search of PubMed, Web of Science, and SCOPUS from inception to September 2025 was performed to capture all possible relevant titles. The keywords and phrases (in different combinations) include Sarcopenia, prevalence of Sarcopenia, Muscle mass, grip strength, gait speed, and the list of all Middle East countries. Boolean operators like “OR” or “AND” were used with search terms. Furthermore, the reference sections of the extracted articles were searched manually to include any articles missed by the electronic search.

### 2.4 Study selection

Studies that met the qualifying requirements were assessed. All research on the prevalence of sarcopenia undertaken in Middle Eastern countries was included in the review. The prevalence rate of sarcopenia was the primary outcome examined. Two authors (BAA and BA) screened suitable studies. A third reviewer (NA) settled disagreements about eligibility.

### 2.5 Data extraction and quality assessment

To extract data, a double-coding strategy was used. A standardized data extraction form was used by two of the investigators (BAAL and AAA) to obtain data. Any issues were discussed until agreement was reached.

The methodological quality and risk of bias of the included studies were independently evaluated using the Newcastle–Ottawa Scale (NOS) adapted for cross-sectional and prevalence studies. The NOS was specifically selected to ensure methodological alignment and direct comparability with landmark global and regional sarcopenia prevalence meta-analyses. This tool assesses three primary methodological domains across eight items: (1) Selection of study participants (representativeness of the sample, sample size justification, non-respondents, and ascertainment of exposure); (2) Comparability of study groups based on design and statistical control for confounding variables; and (3) Outcome assessment (independent/blinded assessment and appropriateness of the statistical test).

The primary outcome was the prevalence of sarcopenia. Most included studies were cross-sectional and assessed sarcopenia at a single time point. Therefore, the reported estimates were interpreted as point prevalence. When the type of prevalence was not explicitly specified, it was inferred based on study design and measurement context.

### 2.6 Data synthesis and analysis

A random-effects meta-analysis with 95% CIs was used to estimate the pooled prevalence. A subgroup analysis of the prevalence was carried out if there were enough studies in each subgroup using meta-regression analysis. Subgroups were divided by gender (Male vs. Female). Potential publication bias across included studies was graphically examined using funnel plots, and funnel plot asymmetry was evaluated using Egger's test. Statistical heterogeneity across studies was assessed using the I^2^ statistic, with higher values indicating greater between-study variability.

It is important to note that the primary aim of this meta-analysis was to provide a broad epidemiological overview of the burden of sarcopenia in the Middle East, where the existing evidence base is limited and methodologically heterogeneous. Given the relatively limited number of available studies and the diversity of diagnostic approaches used across the region, restricting the analysis to a single definition of sarcopenia would have substantially reduced study inclusion and limited regional representativeness. Therefore, all eligible studies were included to maximize coverage while acknowledging the resulting conceptual variability. Accordingly, the pooled prevalence estimate should be interpreted as an aggregated summary reflecting variability in current diagnostic and measurement practices across studies.

## 3 Results

### 3.1 Study selection and characteristics

The search resulted in a total of 1,123 studies (PubMed: 507, Web of Science: 487, and SCOPUS: 129), of which the full text of 26 studies was reviewed, and only 11 studies were eligible to be included in the meta-analyses. Figure 1 shows the selection process for the included studies. The sample sizes in the included studies ranged from 127 to 2,816 participants, with a mean age ranging from 64 to 76 years. Four studies reported sarcopenia using the EWGSOP criteria, and one study used AWGS criteria. Only one study used SARC-F as the diagnostic criterion. Six countries have reported the prevalence of sarcopenia, including Saudi Arabia, Turkey, Lebanon, Egypt, Iran, and Palestine. The total prevalence rate ranged from 5.2% to 64.8% (Table 1).

**Figure 1:**
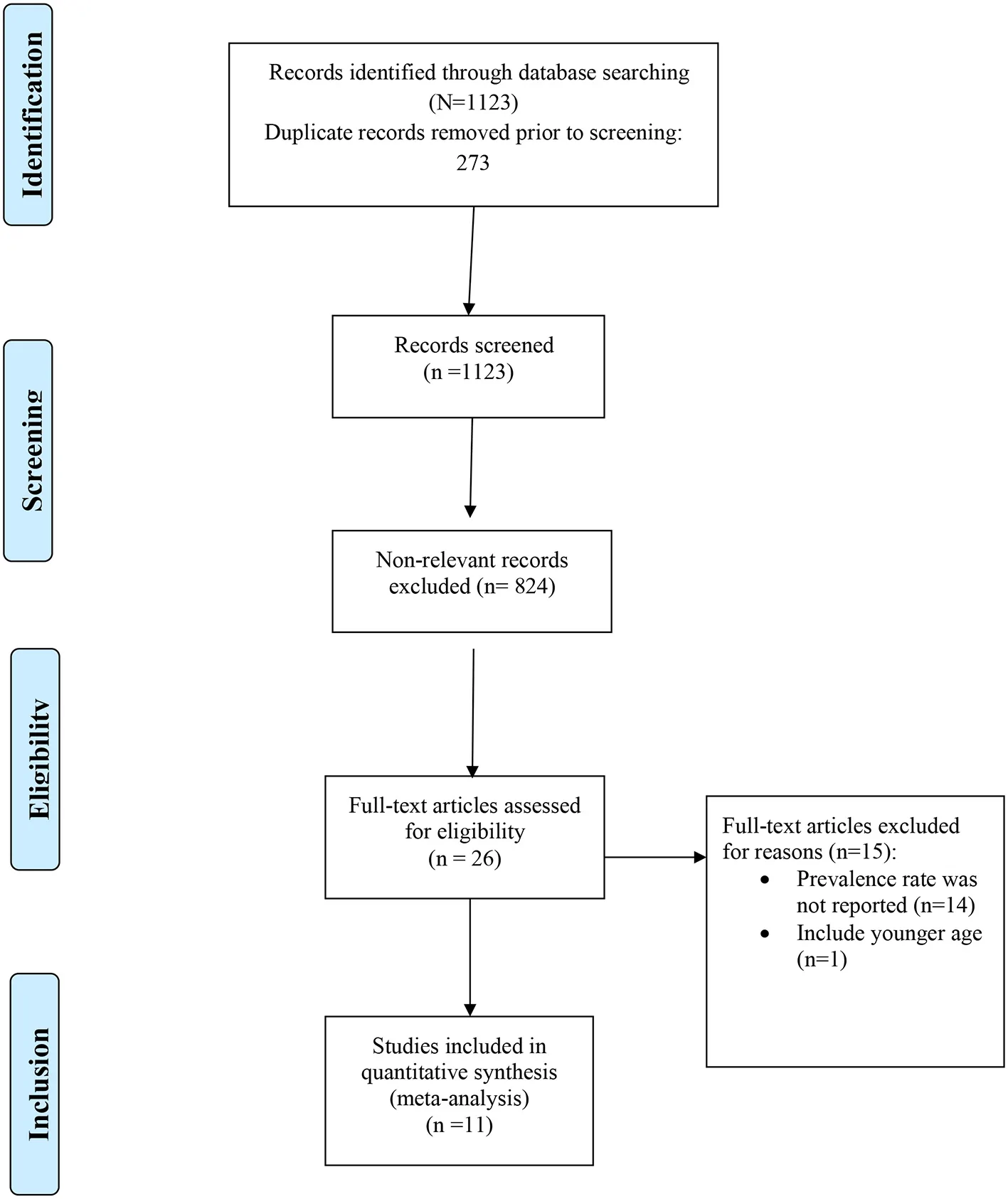
Flowchart of the systematic review and meta-analysis according to the PRISMA guidelines.

**Table 1:** Characteristics of studies included in the systematic review.

Study	Country	Definition of sarcopenia	Assessment of body composition	Muscle strength	Physical performance	Mean age	Sample size (*N*)	Sarcopenia count	Sarcopenia male %	Sarcopenia female %	NOS score
Sanad et al. (43)	Egypt	EWGSOP	BIA	Grip strength	10-m gait speed	64	127	40	47.60%	14.20%	7
Dorosty et al. (34)	Iran	EWGSOP	BIA	Grip strength	Gait speed	70.8	644	207	27.10%	6.60%	7
Saadeddine et al. (35)	Lebanon	Sarcopenia definition based on Oh and colleagues {[appendicular lean mass (ALM)/weight] × 100%}	BIA	NA	NA	67.9	243	67	29.60%	23.40%	6
Badrasawi et al. (36)	Palestine	EWGSOP	BIA	Grip strength	Gait speed	69.5	145	39	34.70%	17.90%	7
Alodhayani (37)	Saudi Arabia	SARC-F	NA	NA	NA	70.0	498	258	12.90%	58.90%	7
Alsaawi et al. (38)	Saudi Arabia	AWGS	BIA	Grip strength	3 m timed up-and-go test (TUG)	65.9	131	26	NA	19.80%	6
Simsek et al. (39)	Turkey	EWGSOP	BIA	Grip strength	Gait speed	72.8	909	47	6.70%	4.10%	5
Sahin et al. (40)	Turkey	SARC-F	NA	NA	NA	73	404	262	18.80%	42.70%	7
Erkoyun and Uçku (41)	Turkey	EWGSOP	BIA	Grip strength	Gait speed	70	254	165			7
Ates Bulut et al. (44)	Turkey	EWGSOP	BIA	Grip strength	Gait speed	76	2816	893			3
Alqahtani et al. (42)	Saudi Arabia	EWGSOP	BIA	grip strength	gait speed	65.5	182	53	35.85%	64.15%	8

NOS, Newcastle-Ottawa Scale.

### 3.2 Quality assessment

The quality of all included studies ranged from 3 to 8 according to the Newcastle–Ottawa scale, with a median of 7.

## 4 Sensitivity analysis

Sen To test the robustness of the pooled prevalence estimate, two separate sensitivity analyses were performed:

1. Exclusion of Low-Quality Study: Excluding the single study with the lowest Newcastle–Ottawa Scale score (*NOS score* < 4) resulted in a pooled sarcopenia prevalence of 34% (95% CI: 22% 47%).
2. Exclusion of SARC-F Study: Excluding the study that relied solely on the SARC-F screening tool (which screens for probable sarcopenia rather than confirming diagnosis) yielded a pooled prevalence of 33% (95% CI: 21% 46%).

Neither sensitivity analysis produced a substantial change from the primary pooled prevalence estimate of 35% (95% CI: 23% 48%), demonstrating that the primary meta-analysis findings are robust to study quality and diagnostic tool variation.

### 4.1 Meta-analysis results

To estimate the pooled prevalence, a random-effects meta-analysis of 11 studies was used. The pooled prevalence rate of sarcopenia among older adults in the Middle East was 35% (95% CI, 23 to 48; Figure 2). For males, the prevalence rate was 26% (95% CI, 17 to 35), while the prevalence of sarcopenia for females was 28% (95% CI, 15 to 41; see Figure 3). Although the prevalence of sarcopenia was slightly higher among women than men, the magnitude of this difference was small and may not be clinically meaningful. The level of heterogeneity shows for total prevalence among older adults people (*I*^2^ = 99.3%); for prevalence among males (*I*^2^ = 97.6); and for prevalence among females (*I*^2^ = 98.8%). The very high levels of heterogeneity observed across analyses indicate substantial between-study variability, suggesting that the pooled estimates should be interpreted with caution.

**Figure 2:**
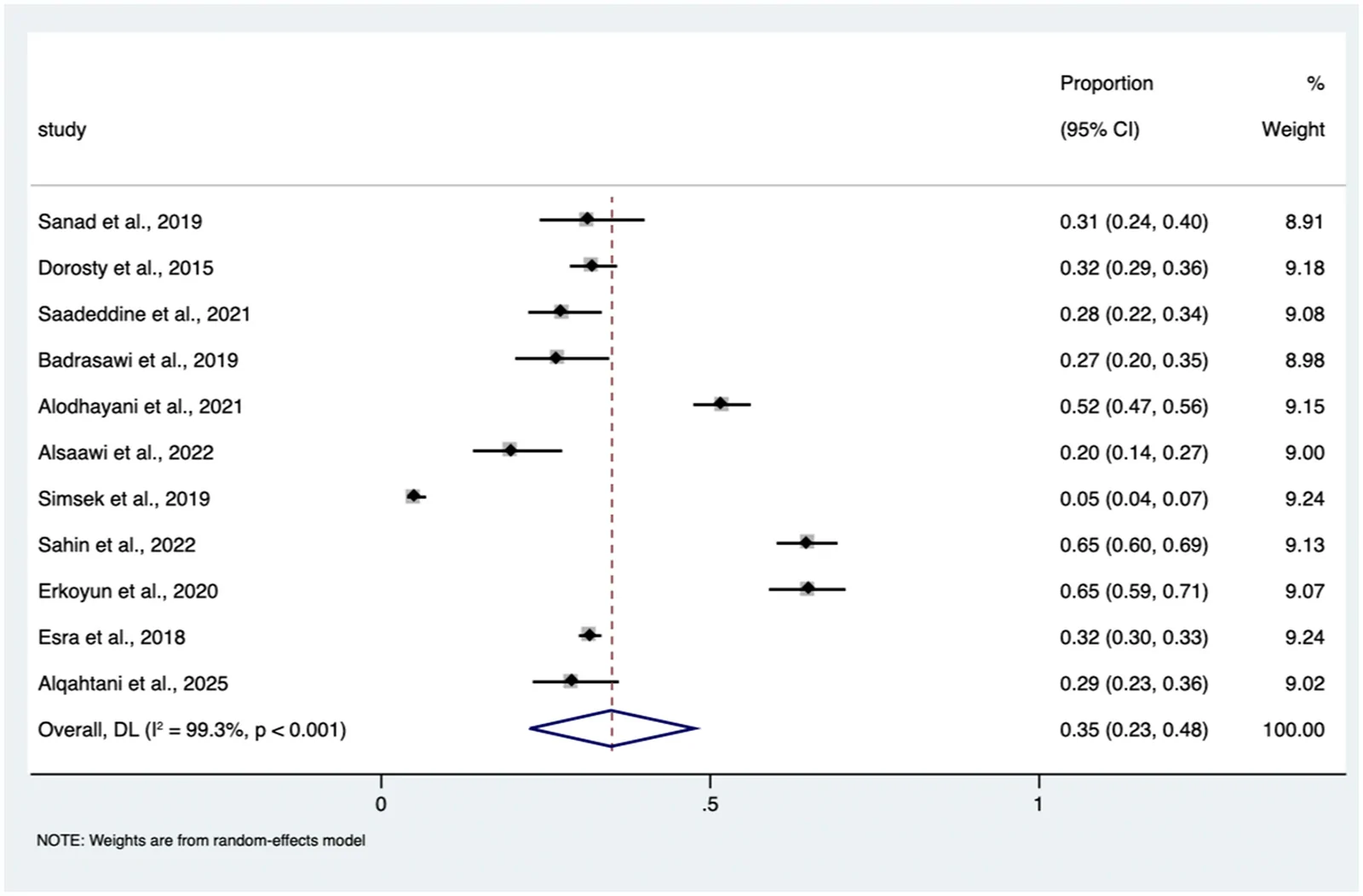
Results of the meta-analysis of sarcopenia prevalence for the total sample.

**Figure 3:**
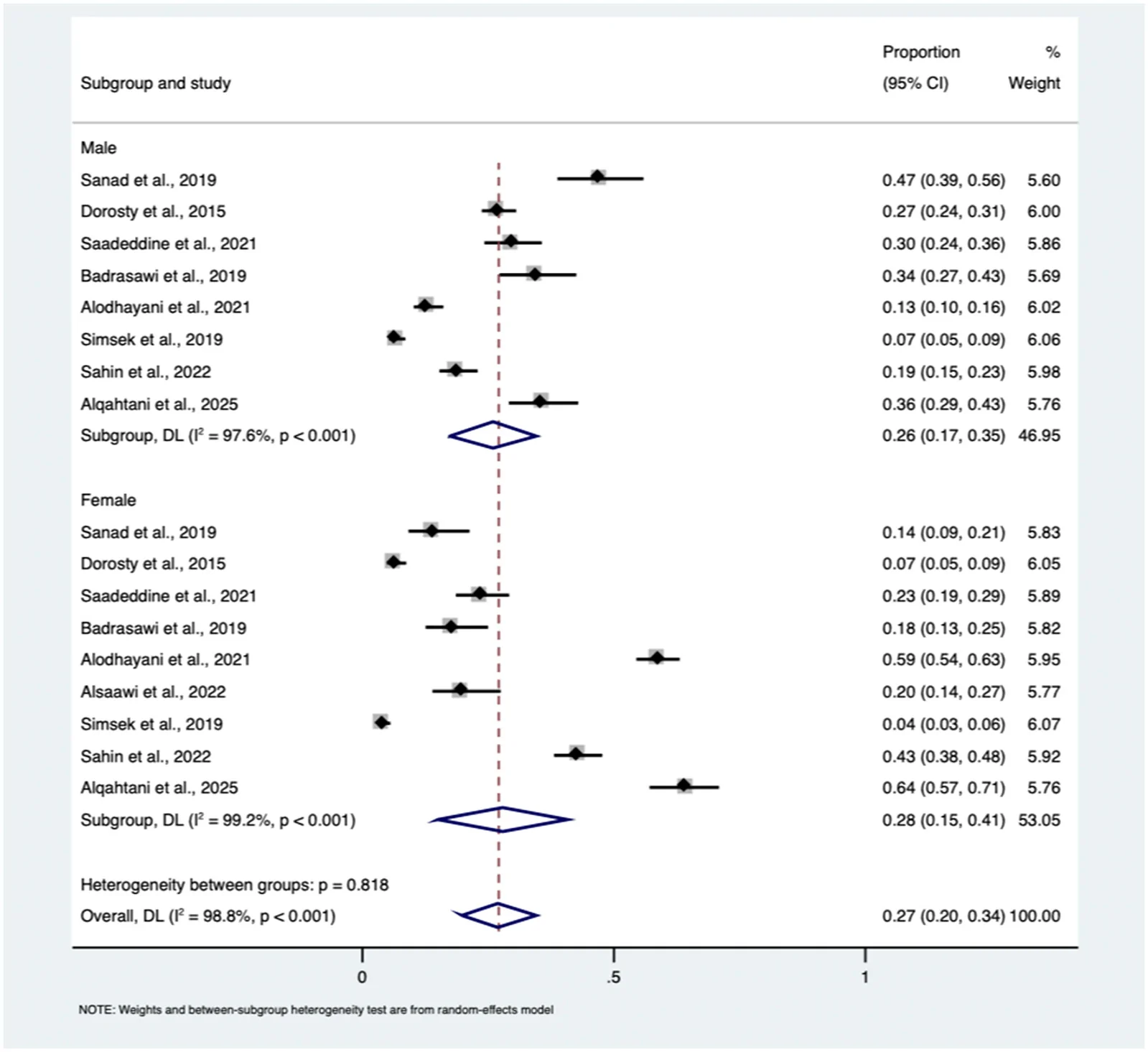
Results of the meta-analysis of sarcopenia prevalence for both males and females.

### 4.2 Publication bias

The funnel plot was visually examined for publication bias. Figure 4 shows the funnel plot for 11 studies. Egger's test (33) was conducted to evaluate the funnel plot asymmetry. Egger's test showed that the funnel plot is not significantly different from symmetry [*t*(9) = −0.15, *p* = 0.880], indicating that the publication bias is minimal (Figure 4). Although Egger's test did not indicate significant publication bias, this finding should be interpreted with caution given the relatively small number of included studies.

**Figure 4:**
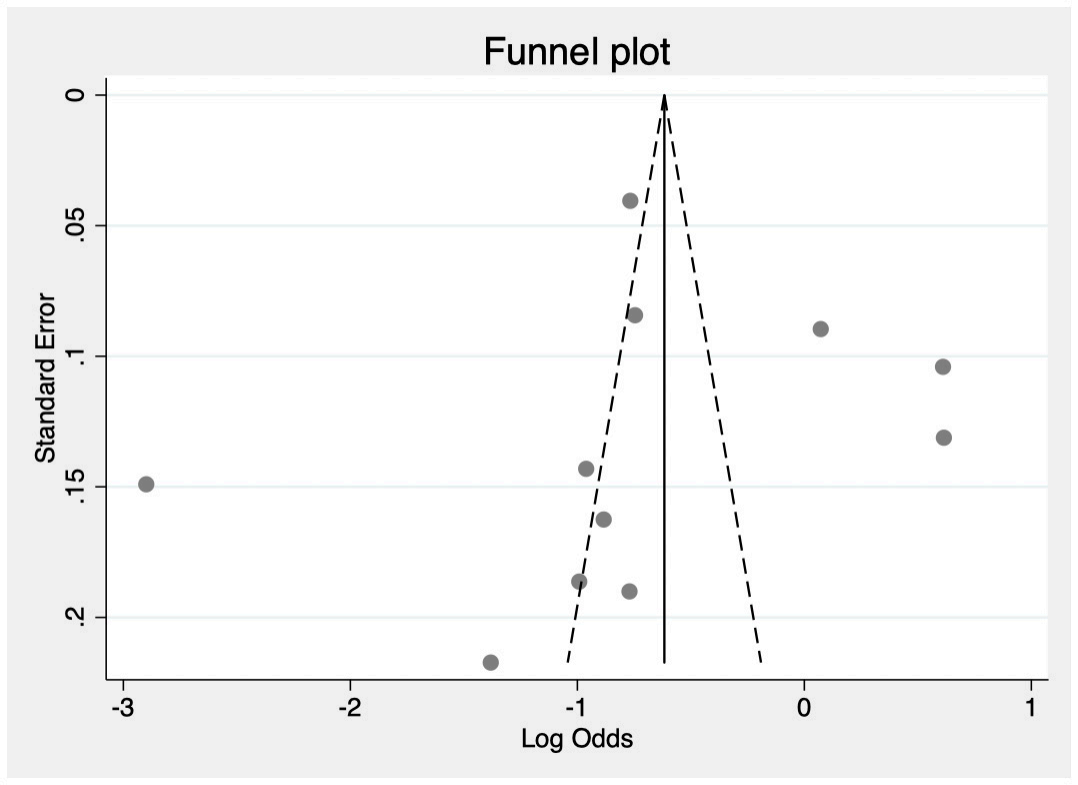
Publication bias.

## 5 Discussion

This meta-analysis aims to determine the prevalence of sarcopenia among healthy community-dwelling older adults in Middle Eastern countries. Eleven studies reported the prevalence of sarcopenia in six countries, and 11 of them were included in the analysis. The included studies used the EWGSOP criteria, the AWGS criteria, and the SARC-F questionnaire for the diagnosis of sarcopenia. The results showed that the overall pooled prevalence of sarcopenia in the Middle East was 35%. There was a significantly higher prevalence of sarcopenia among women (28%) compared to men (26%) in the Middle East countries. The prevalence of sarcopenia in our study (35%) was higher than the global rate, such as in Africa (25%), China (20.7%), and Korea (13.1%) (22–24). This difference can be explained by the variation of different factors such as diet, culture, lifestyle, and ethnically specific genetics among countries and regions.

The most important factors impacting the progression and severity of sarcopenia are levels of physical activity and dietary patterns (25). Individuals with sarcopenia were found to have low appendicular lean mass (ALM) and were less likely to engage in moderate-to-vigorous physical activity (MVPA) (26). A systematic review and meta-analysis described the physical activity (PA) in 20 Middle East and North Africa (MENA) countries reported that the prevalence of PA among the included sample (*n* = 124,702) was 47.9% (27) Another large-scale cross-sectional study among 26000 families in Saudi Arabia reported that only 17.4% of adults meet the minimum recommended physical activity levels (i.e., ≥150 min per week), and those aged ≥65 years old were the lowest practitioners of physical activity (28).

Another factor that could contribute to the progression of sarcopenia is dietary pattern. A cross-sectional study examined the associations between nutrient intake and diagnostic measures of sarcopenia among Arabs and found that individuals with normal muscle mass and high hand-grip strength have greater daily energy from protein and fat intake and a lower percentage of energy from carbohydrates compared to sarcopenic individuals (29).

We found a higher prevalence of sarcopenia among women (28%) compared to men (26%), which is consistent with the results found in China, Japan, and Brazil, but contrasts with those found in Korea and Africa (22–24, 30, 31). Globally, the association between gender and sarcopenia has been inconsistent across the published literature. This inconsistency may reflect cross-country differences in lifestyle patterns and health-related behaviours between men and women. In addition, the higher prevalence of sarcopenia among women could be due to the hormonal changes that accelerate the loss of muscle mass among women compared to men, which becomes obvious following menopause (32). However, these factors were not directly examined in the present meta-analysis and are discussed here only to provide contextual interpretation rather than to imply causal or empirical conclusions.

The high sarcopenia prevalence observed in this review (35%) highlights the need for public health policy and strategy focusing on the assessment of sarcopenia and its modifiable risk factors to promote healthy aging. Clinicians should incorporate objective sarcopenia screening into routine geriatric assessment to inform the design of targeted interventions. A multidisciplinary approach addressing musculoskeletal health, active living, and healthy eating might help in slowing the progression of sarcopenia.

The present study had several limitations. First, this study includes the conceptual heterogeneity in the definition and assessment of sarcopenia across the included studies. The meta-analysis pooled prevalence estimates derived from different approaches, including consensus-based diagnostic criteria (e.g., EWGSOP, AWGS), screening tools such as SARC-F, and surrogate indices. These methods capture related but non-equivalent constructs. Consequently, the overall pooled prevalence estimate should be interpreted with caution, as it represents a synthesis of conceptually heterogeneous measures rather than a single clinically unified definition. This heterogeneity could contribute to the substantial variability in prevalence estimates across studies and may affect the interpretability and clinical applicability of the findings. Second, although this review aimed to determine the prevalence of sarcopenia among healthy community-dwelling older adults in Middle East countries, only six countries had available data, and the other remaining countries were not represented due to the lack of published evidence. Third, the meta-analysis demonstrated extremely high heterogeneity across studies (*I*^2^ > 97%), indicating substantial between-study variability that limits the interpretability of the pooled estimates. This heterogeneity is likely driven by multiple factors, including differences in diagnostic criteria (e.g., EWGSOP, AWGS, SARC-F, and surrogate definitions), variations in population characteristics (e.g., age distribution, sex composition, and health status), study settings, and data collection periods. Such variability is expected in prevalence studies of sarcopenia, given the absence of a universally applied diagnostic standard across regions. Although the diagnostic criteria for sarcopenia varied substantially across studies, subgroup or sensitivity analyses by diagnostic method were not conducted due to the limited number of studies within each category, which may contribute to variability in the pooled estimates. Future research should prioritize the use of standardized diagnostic criteria and sufficiently powered datasets to enable more rigorous investigation of heterogeneity in sarcopenia prevalence estimates. Finally, another factor that may contribute to variability in the prevalence estimates is the wide range of survey years across the included studies. Differences in study periods may reflect underlying secular trends and cohort effects, including population aging and changes in diagnostic criteria for sarcopenia. Given the limited number of studies and variability in reporting, a formal examination of temporal trends or cohort effects was not feasible. However, these factors should be considered when interpreting the pooled prevalence estimates.

Despite limitations, the present study has a number of strengths. First, this study is one of the first to estimate the prevalence of sarcopenia in the Middle East. The data obtained provided comprehensive insight into the prevalence of sarcopenia across six countries and highlighted the need for national-level collaboration to investigate the risk factors of sarcopenia and advance healthy ageing initiatives.

Future research should explore the risk factors of sarcopenia among older populations in the Middle East using both longitudinal and interventional study designs. Large-scale community-based studies should be conducted with standardized diagnostic criteria to estimate sarcopenia prevalence in countries where data are still unavailable. Additionally, well-designed cohort studies are necessary to examine the complex relationships between lifestyle behaviours, dietary patterns, and musculoskeletal health within culturally relevant Middle Eastern contexts.

## 6 Conclusion

This meta-analysis shows that the overall prevalence of sarcopenia in older adults in the Middle East is relatively high (35%), with a higher sarcopenia rate among women (28%) compared to men (26%). Although the prevalence was slightly higher among women than men, this difference was modest and should be interpreted with caution. Nonetheless, the findings suggest that sarcopenia may represent a potential public health concern in the Middle East (however, this interpretation should be made cautiously given the substantial heterogeneity across studies and the limited number of countries represented). These findings highlight the need for further research to better understand the burden of sarcopenia in the region. Future research should include large-scale community-based studies using standardised diagnostic criteria to assess sarcopenia prevalence and investigate the correlation between sarcopenia and possible risk factors such as diet, and musculoskeletal health among older adults in the Middle East.

## Data Availability

The original contributions presented in the study are included in the article/supplementary material, further inquiries can be directed to the corresponding author.
